# Transcriptional Tuning: How Auxin Strikes Unique Chords in Gene Regulation

**DOI:** 10.1111/ppl.70229

**Published:** 2025-04-29

**Authors:** Joseph S. Taylor, Bastiaan O. R. Bargmann

**Affiliations:** ^1^ Virginia Tech School of Plant and Environmental Sciences Blacksburg VA USA

## Abstract

Auxin is a central regulator of plant growth, development, and responses to environmental cues. How a single phytohormone mediates such a diverse array of developmental responses has remained a longstanding question in plant biology. Somehow, perception of the same auxin signal can lead to divergent responses in different organs, tissues, and cell types. These responses are primarily mediated by the nuclear auxin signaling pathway, composed of ARF transcription factors, Aux/IAA repressors, and TIR1/AFB auxin receptors, which act together to regulate auxin‐dependent transcriptional changes. Transcriptional specificity likely arises through the functional diversity within these signaling components, forming many coordinated regulatory layers to generate unique transcriptional outputs. These layers include differential binding affinities for *cis*‐regulatory elements, protein–protein interaction‐specificity, subcellular localization, co‐expression patterns, and protein turnover. In this review, we explore the experimental evidence of functional diversity within auxin signaling machinery and discuss how these differences could contribute to transcriptional output specificity.

## 1 INTRODUCTION

The phytohormone auxin plays a crucial role in nearly all aspects of plant growth, development, and responses to environmental cues (reviewed in Woodward and Bartel 2005). One of the longstanding questions in plant biology is how this simple molecule can elicit so many different cellular responses, depending on where and when it is perceived. For instance, asymmetric redistribution of local auxin concentrations is the common basis of many tropic responses; however, a higher concentration on the shady side of the hypocotyl leads to a promotion of cell expansion during phototropism, whereas a higher concentration of auxin on the bottom side of the root apical meristem leads to an inhibition of cell expansion during gravitropism (reviewed in Muday 2001).

Cellular responses to altered auxin concentrations are predominantly mediated by transcriptomic changes through the canonical nuclear auxin signaling pathway, although certain rapid responses, such as membrane depolarization and cell‐wall acidification, have been shown to be independent of the transcriptional output of this pathway (Yu et al. 2022; Lin et al. 2023). The canonical nuclear auxin signaling pathway is composed of three components: the TRANSPORT INHIBITOR RESPONSE 1 (TIR1)/AUXIN‐SIGNALING F‐BOX (AFB) auxin receptors, the AUXIN/INDOLE‐3‐ACETIC ACID (Aux/IAA) transcriptional repressors, and the AUXIN RESPONSE FACTOR (ARF) transcription factors (Lokerse and Weijers 2009). In flowering plants, each of these components is encoded by sizeable gene families: six TIR1/AFBs, 29 Aux/IAAs, and 23 ARFs in the model plant *Arabidopsis thaliana* (Dharmasiri et al. 2005; Okushima et al. 2005; Overvoorde et al. 2005).

The importance of this pathway in the global auxin response is demonstrated by the fact that several components were identified in various screens for auxin insensitivity, e.g., TIR1 was found in a screen for mutants resistant to the auxin transport inhibitor Naphthylphthalamic acid and IAA17/AUXIN RESISTANT 3 (AXR3) was found in a screen for mutants resistant to the synthetic auxin 2,4‐Dichlorophenoxyacetic acid (Leyser et al. 1996; Ruegger et al. 1998). The ARF transcription factors tend to have more specific phenotypes, e.g., ARF7/NON‐PHOTOTROPIC HYPOCOTYL 4 (NPH4) was identified in a screen for phototropic mutants and ARF5/MONOPTEROS (MP) was identified because it lacks embryonic root formation (Berleth and Jürgens 1993; Liscum and Briggs 1995).

Changes in auxin concentration can affect the transcription of thousands of genes throughout the plant (Hagen et al. 1984; Lewis et al. 2013). Many of these transcriptomic changes are dependent on the canonical nuclear auxin signaling pathway, e.g., about half of the auxin‐responsive genes show impaired auxin‐mediated regulation in the *axr3‐1* mutant (Overvoorde et al. 2005). It seems likely that context‐dependent differences in the auxin response are based on differences in gene expression responses. Indeed, auxin‐responsive transcriptomic changes vary depending on the organ, tissue, or cell type examined (Gee et al. 1991; Paponov et al. 2008; Bargmann et al. 2013).

It is hypothesized that the specific transcriptional response of a particular cell to an auxin signal is determined by which components of the signaling pathway are expressed in that cell. In concordance with this putative mechanism, the different gene family members encoding the TIR1/AFB, Aux/IAA, and ARF signaling components show varying spatial and temporal expression patterns (Rademacher et al. 2011; Piya et al. 2014; Prigge et al. 2020; Truskina et al. 2020). However, specific evidence to substantiate this hypothesis is challenging to interpret due to the convoluted and promiscuous interaction network formed by this seemingly simple yet practically intricate signaling pathway.

In addition to transcriptional regulation, auxin also drives non‐transcriptional signaling to regulate rapid cellular responses. These alternative pathways, though not the focus of this review, play important roles in auxin‐mediated processes. For example, auxin influx carrier AUX1, auxin receptor AFB1, and calcium channel CNCG14 act together to enable fast cytosolic calcium influx, mediating fast auxin signaling in roots (Dindas et al. 2018). Moreover, the most notable player in non‐canonical auxin signaling is AUXIN‐BINDING PROTEIN 1 (ABP1), identified by Hertel et al. (1972). Apoplastic ABP1 interacts with transmembrane kinase TMK1, which act together to enable auxin‐dependent responses. Auxin perception by ABP1‐TMK1 triggers the phosphorylation of thousands of proteins and is crucial for rapid cellular responses to auxin (such as H + ‐ATPase activation and cytoplasmic streaming). It is also involved in the formation of auxin transport channels and regeneration of vascular tissues after wounding (Friml et al. 2022). Recently, additional auxin‐binding proteins (ABL1 and ABL2) also interacting with TMK1 have been identified (Yu et al. 2023). While these mechanisms act on timescales much faster than transcriptional auxin responses, whether and how the different auxin signaling pathways interact requires further research. In this review, we will focus on how auxin enables transcriptional specificity through the nuclear auxin signaling pathway.

**FIGURE 1:**
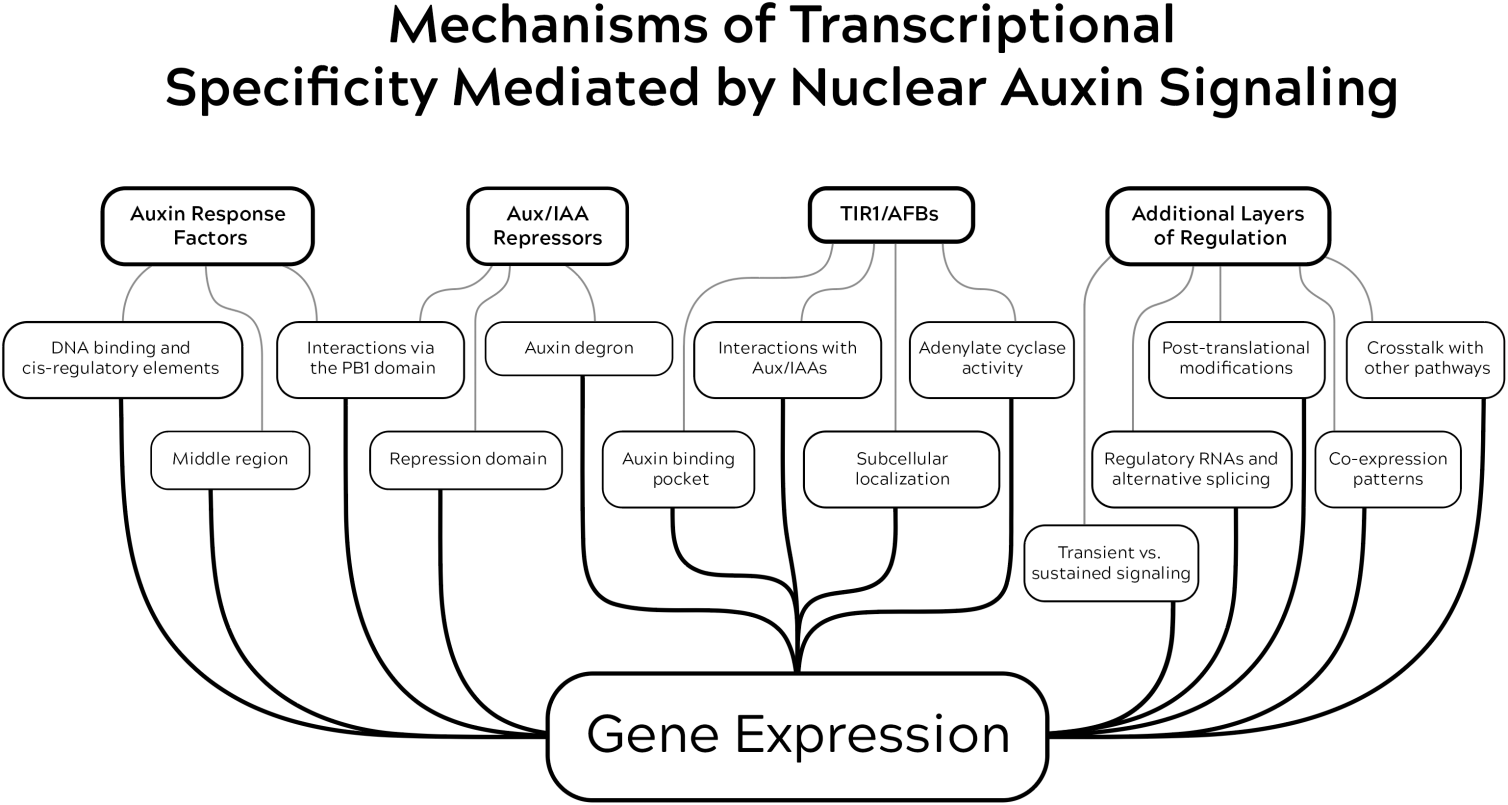
Diagram of the layers of regulation that coordinate auxin‐mediated gene expression.

Here, we explore the experiments that have contributed to our current understanding of the mechanisms underlying auxin's profound ability to regulate so many different aspects of plant growth. After summarizing the components of the nuclear auxin signaling pathway, we will systematically dissect the potential mechanisms of transcriptional specificity mediated by auxin, focusing on the functional diversity within the pathway (Figure 1). We will navigate through the pathway from the bottom up, starting with ARF transcription factors and DNA binding, transitioning to Aux/IAA repressors and the TIR1/AFB receptors. We will then briefly discuss additional layers of regulation that may also contribute to signaling specificity, such as hormone crosstalk, regulatory RNAs, alternative splicing, co‐expression patterns, post‐translational modifications, and non‐canonical auxin signaling. Finally, we will conclude our discussion by speculating where we might go from here to further our understanding of specificity in the auxin signaling‐induced transcriptomic output. Unless otherwise specified, this discussion will focus on auxin signaling in *Arabidopsis thaliana* for simplicity.

## 2 THE CANONICAL NUCLEAR AUXIN SIGNALING PATHWAY

**FIGURE 2:**
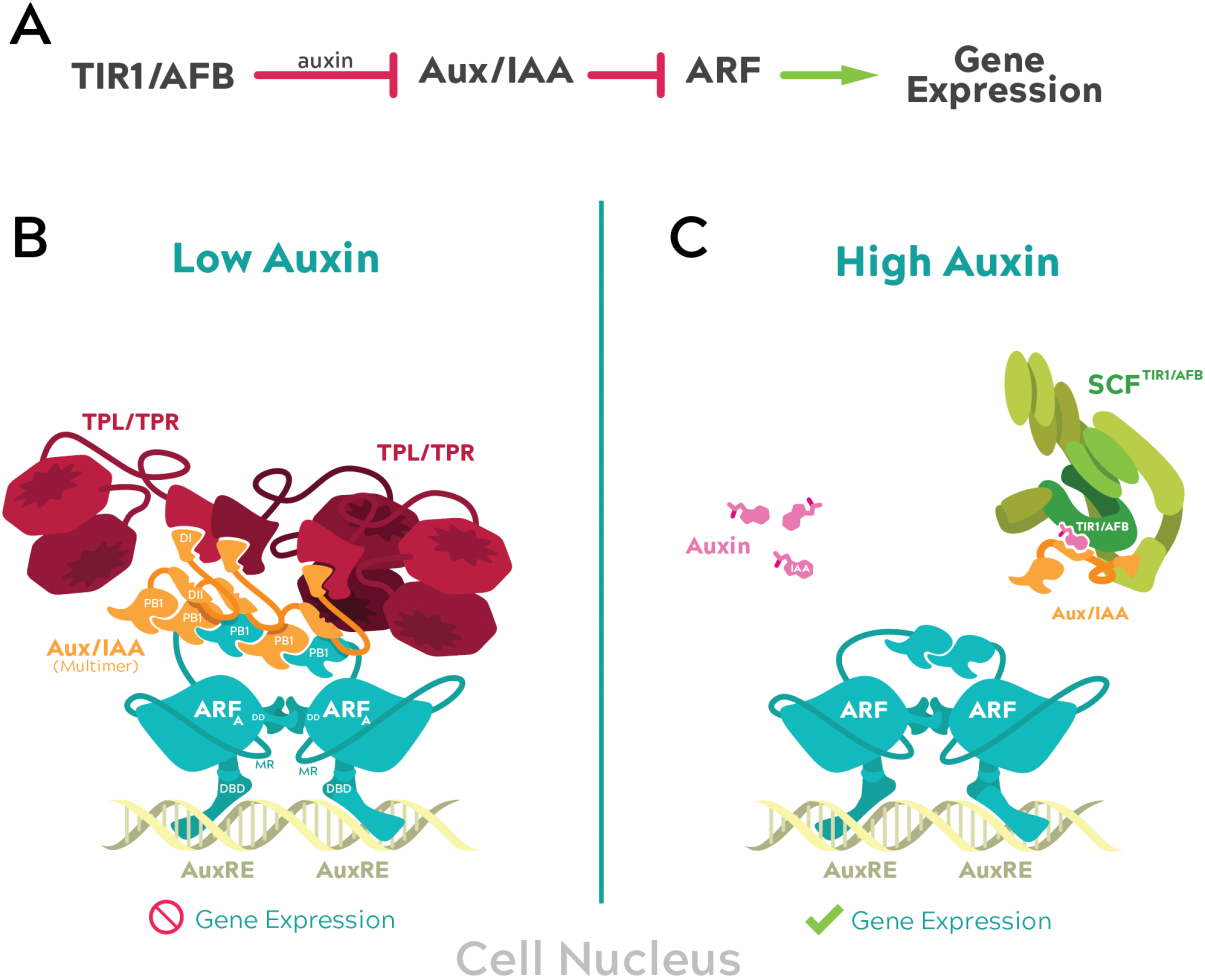
Schematic and illustrated representations of the nuclear auxin signaling pathway. A) Schematic diagram of the nuclear auxin signaling pathway. B) Under low auxin concentrations in the cell nucleus, gene expression is blocked. Activator ARF protein dimers (teal) are bound to auxin response element (AuxRE) sequences. Aux/IAA repressors (orange) multimerize on the C‐terminal PB1 domain of ARF proteins and recruit TPL/TPR proteins (red), inducing chromatin condensation. Functional domains for ARF [DNA binding domain (DBD), dimerization domain (DD), middle region (MR), and PB1 domain] and Aux/IAA proteins (Domain I (DI), Domain II (DII), and PB1 domain) are labeled. C) Under high auxin concentrations (pink), Aux/IAA proteins interact with the SCF^TIR1/AFB^ complex (green) in an auxin‐dependent manner. ARF proteins, relieved from repression, activate auxin‐responsive gene expression.

The canonical nuclear auxin signaling pathway is composed of three components: the TIR1/AFB receptors, the Aux/IAA repressors, and the ARF transcription factors (Figure 2A). When auxin concentration is low in the cell nucleus, transcriptional activation by ARF transcription factors is inhibited by interactions with Aux/IAA repressors (Ulmasov et al. 1997b). This interaction is facilitated by PHOX AND BEM1 (PB1) protein interaction domains (reviewed in Guilfoyle and Hagen 2012). Aux/IAA proteins recruit and interact with co‐repressors TOPLESS (TPL) and TPL‐RELATED (TPR) (Figure 2B), resulting in chromatin condensation and transcriptional inactivation (Szemenyei et al. 2008; Krogan et al. 2012b). As nuclear auxin concentration increases, Aux/IAA proteins complex with the SCF^TIR1/AFB^ (S‐PHASE KINASE‐ASSOCIATED PROTEIN 1‐CULLIN‐F‐BOX) E3 ubiquitin ligase through the TIR1/AFB F‐box proteins in an auxin‐dependent manner. Aux/IAA proteins are polyubiquitylated by the SCF^TIR1/AFB^ complex and subsequently degraded (Figure 2C). Free from the repression of Aux/IAAs, ARF proteins recruit chromatin remodeling machinery and RNA polymerase activity, and trigger gene expression (Wu et al. 2015).

Plant genomes encode all three modular signaling components—ARFs, Aux/IAAs, and TIR1/AFBs—through multi‐member gene families, e.g., in Arabidopsis: 23 ARFs, 29 Aux/IAAs, and six TIR1/AFBs and in soybean: 55 ARFs, 61 Aux/IAAs, and 14 TIR1/AFBs (Ferreira Neres et al. 2024). The conservation of these large gene families suggests a degree of functional specialization. Indeed, mutation of homologous genes within the same genome can lead to divergent phenotypes. For example, while a gain‐of‐function Aux/IAA mutation, *iaa12‐1/bodenlos* (*bdl*), and loss‐of‐function ARF mutation, *arf5‐1/mp*, both result in a lack of embryonic root development (Berleth and Jürgens 1993; Hamann et al. 1999), another gain‐of‐function Aux/IAA mutation, *iaa14‐1/solitary root* (*slr*), and loss‐of‐function ARF double mutation, *nph4‐1/arf19‐1*, both lead to impaired lateral root development (Fukaki et al. 2002; Okushima et al. 2005). These functional differences among ARFs and Aux/IAAs appear to be partially due to protein sequence variations, as demonstrated by misexpression and promoter‐swap studies (Weijers et al. 2005; Rademacher et al. 2012; Lavy et al. 2016). In the following sections, we will explore how functional differences between members of the three‐core nuclear auxin signaling pathway components and their interactions can contribute to specificity in signaling output.

## 3 ARF TRANSCRIPTION FACTORS

Multi‐member *ARF* gene families are present throughout flowering plants and are divided into four distinct phylogenetic groups, referred to as class A, B, C, and D (Finet et al. 2013; Bascom et al. 2023). Class A ARF proteins are the most extensively studied subfamily, have relatively low diversity, and function as transcriptional activators. Class B ARFs are highly divergent and are thought to function as transcriptional repressors. Class C ARFs may also function as transcriptional repressors, yet recent work questions their involvement in auxin signaling (Flores‐Sandoval et al. 2018; Kato et al. 2020). Finally, a sister clade of class A, class D ARFs have recently been designated in non‐vascular plants but are absent in tracheophytes (Bascom et al., 2023).

**FIGURE 3:**
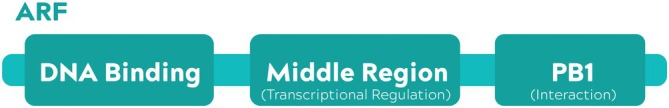
Schematic of the three functional domains found in canonical ARFs. N‐terminal DNA‐binding domain: binds AuxRE sequences and promotes formation of ARF dimers; middle region: confers transcriptional activation or repression; C‐terminal PB1 domain: confers interactions with Aux/IAA repressors and other ARFs.

Aside from class D ARFs, which lack the DNA binding domain, ARF proteins typically have three functional domains (Figure 3): a N‐terminal DNA binding domain, a middle region that confers activation or repression depending on ARF class, and a C‐terminal PB1 protein interaction domain (Tiwari et al. 2003; Korasick et al. 2014; Nanao et al. 2014). Recent work examining the specific roles of these domains offers insight into how they work together to contribute to the diversity of auxin signaling specificity.

### 3.1 The ARF DNA‐Binding Domain

Transcriptional specificity has been suggested to be predominantly mediated by the affinity of individual ARF proteins for distinct regulatory targets. ARF proteins interact with the promoters of genes they regulate through the DNA‐binding domain. This domain has been shown to consist of three components through structural analysis: a two‐part dimerization domain, a B3 subdomain embedded within the dimerization domain and required for DNA binding, and a Tudor‐like ancillary domain whose function is currently unknown (Guilfoyle et al. 1998; Ulmasov et al. 1999a; Boer et al. 2014). These structures likely play a role in signaling specificity as they regulate which gene promoters individual ARFs can bind.

ARFs bind to repeats of six‐base pair TGTC‐containing motifs referred to as auxin response elements (AuxREs) (Ulmasov et al. 1997a). The commonly identified AuxRE (TGTCTC) was first described through “promoter bashing” in soybean (Liu et al. 1994). Many ARFs have been shown to bind this motif *in vitro* using yeast‐1‐hybrid, gel mobility shift, and X‐ray crystallography assays (Ulmasov et al. 1997a, 1999a; Boer et al. 2014). Crystallography of ARF1 and ARF5/MP in complex with this motif uncovered the structural basis for this interaction and identified key residues involved (Boer et al. 2014). The DNA‐contacting residues of the B3 subdomain are highly conserved across the ARF protein family, which calls into question how distinct genes can be targeted by specific ARFs.

Later studies have suggested that ARFs may target distinct AuxREs separated by class. Crystal structures, DAP‐seq, and computational analyses have suggested that ARFs may have different affinities for variations in the last two nucleotides of the TGTC motif (TGTCNN), but it remains unclear if these differences hold up *in vivo* (O'Malley et al. 2016; Zemlyanskaya et al. 2016; Galli et al. 2018; Lieberman‐Lazarovich et al. 2019; Freire‐Rios et al. 2020). DAP‐seq, when used to analyze the DNA‐binding landscape of ARFs in maize, uncovered distinct binding targets between class A and class B ARFs (Galli et al. 2018). While there was substantial overlap between class A and B binding peaks, there also was a significant number of sites bound by only one class of ARF. Class A ARFs were found to preferentially bind TGTCGG motifs, while class B ARFs preferred AuxREs with a cytosine‐rich tail (i.e., TGTCCCCC). Interestingly, when assaying maize ARFs binding to the Arabidopsis genome or removing reads from highly repetitive regions in the maize genome, the distinction between classes became insignificant and all ARFs were similarly enriched for TGTCGG or TGTCNN motifs. Open chromatin profiling (ATAC‐seq) revealed tissue‐specific ARF binding for AuxRE sequences (Galli et al. 2018), which suggests that genome accessibility can influence ARF binding affinity. On the whole, the maize study supports previous evidence (Boer et al. 2014) that there is relatively little preference for distinct AuxREs within a particular ARF class.

Class B repressor ARFs have been hypothesized to act antagonistically to class A ARFs by competing for binding sites or via heterodimerization. In support of the competitive binding model, crystal structures have revealed that ARF1 (class B) and ARF5/MP (class A) both bind TGTCGG preferentially over the TGTCTC AuxRE (Boer et al. 2014; Freire‐Rios et al. 2020). Overlapping affinities of ARFs, both within and across ARF classes, for the same AuxRE sequences (*in vitro*) contradicts the notion that AuxRE binding alone drives the transcriptional specificity of auxin signaling.

In contrast with the DNA binding similarities observed in class A and B ARFs, the DNA binding domain in class C ARFs appears to have diverged functionally. Recent domain swap experiments in *Marchantia polymorpha* have illustrated that ARF chimeras generated by swapping DNA‐binding domain of MpARF3 (class C) with that from MpARF1 (class A) or MpARF2 (class B) were unable to rescue the *Mparf3* mutant (Hernández‐Garcia et al. 2024). Interestingly, ARF chimeras generated by swapping the middle region or PB1 domain while retaining the MpARF3 DNA binding domain were able to complement the mutant to varying degrees. This suggests that the DNA binding domain serves as the main region of functional specialization in class C ARFs, which may otherwise functionally overlap with class A and B ARFs (Hernández‐Garcia et al. 2024). These differences in DNA binding likely mediate unique transcriptional responses, but more research is necessary to clarify any role class C ARFs play in auxin signaling.

The overlap in DNA binding between and within class A and B ARFs ultimately lacks the nuance to explain the observed diversity in auxin signaling specificity. It has been demonstrated that AuxRE number, spacing, and orientation are intimately tied to ARF binding affinity and activation of gene expression. For example, a study that employed a minimal auxin circuit in yeast revealed that most class A ARFs require at least two adjacent AuxREs to activate gene expression (Pierre‐Jerome et al. 2016). Moreover, this study showed that mutating specific residues in the dimerization domain within the DNA‐binding domain causes a complete loss of gene activation. This supports previous crystallography evidence demonstrating that ARF's DNA‐binding domains consistently homodimerize (Boer et al. 2014). This same work also pointed out that, while most of the ARF DNA‐binding domain is highly conserved, most sequence variations fall within the loops that connect the B3 and dimerization domains. The authors hypothesized that this less conserved region could contribute to distinct ARF dimer preferences for AuxRE repeat spacing. Structural analysis demonstrated that ARF1 and ARF5/MP can bind inverted AuxRE repeats (e.g., TGTCTC‐spacer‐GAGACA) separated by seven to eight nucleotides. Moreover, ARF5/MP dimers seem to exhibit more flexibility in AuxRE spacing than ARF1 dimers. This finding formed the foundation of the “molecular caliper model,” which proposes that different ARFs bind AuxREs as dimers with distinct affinities for their orientation and spacing (Boer et al. 2014).

Building on the structural and yeast studies, DAP‐seq analyses have further clarified the specific binding rules that govern ARF‐AuxRE binding. ARFs dimers bind adjacent AuxREs in three distinct configurations: direct repeats (DR; AuxREs following each other), inverted repeats (IR; in a palindromic configuration, pointing toward each other), and everted repeats (ER; in a palindromic configuration, facing away from each other). Unique AuxRE spacing preferences were detected for each orientation, with more flexibility awarded by class A ARFs (IR7–8 and IR18; DR 4–5, DR14–16, and DR25; and ER3, ER13 and ER23) compared to the class B ARFs (IR7–8) (O'Malley et al. 2016; Galli et al. 2018; Stigliani et al. 2019) (reviewed in Cancé et al. 2022). Freire‐Rios et al. (2020) identified connections between AuxRE orientation and its association with activation or repression using qRT‐PCR, RNA‐seq, and microarray analyses. IR8 was associated with activation and repression depending on the context, while DR5 was only associated with activation (Freire‐Rios et al. 2020).

Structural analysis has suggested that class B ARFs may preferentially bind to AuxRE sites in the IR orientation (Boer et al. 2014), while studies in yeast have shown that class A ARFs bind all three AuxRE‐repeat configurations but activate most strongly in the IR configuration (Lanctot et al. 2020). ARF19 was the only class A ARF that could activate gene expression by binding a single AuxRE and did so almost as strongly as promoters with two AuxREs. However, ARF19 dimerization was still required for activation and the unique ability to bind single AuxREs was not observed with the ARF19 ortholog, ZmARF27 (Lanctot et al. 2020). Orientation relative to the transcription start site is also important. ARF19 was the only class A ARF reported by Lanctot et al. (2020) that could activate gene expression on direct AuxRE repeats facing away from the transcription start site. This serves as evidence of AuxRE configuration contributing to transcriptional specificity by specific ARF dimers.

While previous studies have elucidated the structural elements and relevant interactions associated with ARF DNA‐binding, they have been primarily limited to qualitative methods. For example, ARF dimerization is required for gene expression, but quantitative data on the kinetics of these interactions and their effect on DNA‐binding affinity has been limited. Additionally, the PB1 domain has been implicated in the stability of ARF dimers as its removal or mutations significantly reduces ARF activation of reporter gene expression in yeast (Pierre‐Jerome et al. 2016; Lanctot et al. 2020). Moreover, class A and B ARFs lacking the PB1 domain were not able to complement *arf* mutants in *M. polymorpha*, which may be due to reduced dimer stability. Substituting the PB1 domain with an oligomerization domain from the LEAFY transcription factor partially rescued the ability to complement mutants in *M. polymorpha* (Kato et al. 2020).

More recently, Fontana et al. (2023) employed single‐molecule Förster Resonance Energy Transfer (smFRET) to provide a quantitative view of ARF DNA‐binding. This study highlighted the importance of cooperative action between ARF DNA‐binding and PB1 domains to promote high‐affinity binding to AuxREs. The ARF2 DNA‐binding domain was found to be sufficient to bind DNA, but the presence of the PB1 domain increases the affinity of DNA binding through dimer stability. A four‐state cyclic model was developed to quantify the affinity and kinetics of ARF interaction with the tested composite AuxRE (IR7). The model revealed that the increase in affinity from the presence of the PB1 domain could be completely attributed to a shift in the dimer–monomer equilibrium. Analysis of additional ARFs showed that changes in dimer stability induced by variations in the DNA binding domain exhibit the same pattern of dimerization kinetics as those caused by changes in the PB1 domain (Fontana et al. 2023). This work demonstrates that ARF binding affinity for AuxRE repeats is largely conferred by the stability of ARF dimers, regardless of the source of that stability, and serves as a key factor in ARF‐mediated transcriptional regulation. However, bioinformatic scans have suggested that closely spaced AuxREs are rather rare in the Arabidopsis genome and often deviate from the optimal sequence or orientation (Grigolon et al. 2018). This suggests that other factors must also play a role in regulating auxin signaling specificity.

The rarity of “ideal” AuxRE syntax in auxin‐responsive promoters could point to the involvement of other transcription factors contributing to auxin response, a model supported by the prevalence of other transcription factor binding sites within these promoters (Berendzen et al. 2012; Mironova et al. 2014; Cherenkov et al. 2018). Ulmasov et al. (1995) reported that AuxREs are essential but not sufficient for driving auxin response. This study revealed that, in addition to the TGTCTC motif, there were additional upstream sequences required for auxin inducibility. These sequences were required for gene expression and exhibited constitutive activity when the AuxRE was mutated or deleted. This indicated that at least some AuxREs have a larger composite structure that consists of a constitutive element and an adjacent AuxRE, which confers auxin inducibility (Ulmasov et al. 1995).

More recently, Novikova et al. (2024) showed that these composite structures contained transcription factor binding sites. They mined the Arabidopsis genome and identified coupling elements adjacent to AuxREs in auxin‐responsive genes that resembled transcription factor binding sites. These coupling elements are positioned within 15 bp of AuxRE sites to form a composite, bipartite AuxRE (biAuxRE) with the following structure: TGTCNN‐spacer‐NNNNNN or NNNNNN‐spacer‐TGTCNN. 80% of the biAuxREs were found in early auxin‐responsive genes, with nearly a three‐fold bias towards activation over repression (Novikova et al. 2024). Validated against canonical AuxRE repeats in known auxin‐responsive genes, this study identified a previously unknown configuration (DR1), which was the most abundant repeat associated with auxin‐dependent gene activation. Interestingly, previously undescribed ER12 and IR14 were the only elements associated with auxin‐induced repression. Several of the identified biAuxREs were functionally validated *in vivo* by mutating them in the context of a full promoter driving GFP and visualized in auxin‐treated roots.

Novikova et al. (2024) went on to identify transcription factor families associated with binding to the identified coupling elements of biAuxREs. A significant number of coupling elements matched known transcription factor binding sites, with motifs associated with AP2/ERF, bZIP, C2H2, HD‐ZIP, MYB, TCP, and WRKY families appearing most frequently. A combination of biAuxRE mutagenesis, study of DNA‐protein and protein–protein interactions, and root phenotypes enabled the development of a model for biAuxRE regulation of *IAA30* expression. The model suggests that *IAA30* is, at least in part, co‐regulated by a biAuxRE attenuated by WRKY7 and ARF5/MP dimers, which work together to activate gene expression (Novikova et al. 2024). These findings emphasize that ARF‐cofactors recruited to AuxRE coupling elements play important roles in regulating ARF activity at the sites ARF binds.

Furthermore, a recent exploration of the evolutionary origins and functional diversification of ARFs have highlighted the enigmatic Tudor‐like ancillary domain within the DNA binding domain. (Hernández‐Garcia et al. 2024). The ARF dimerization and ancillary domains form a fold with homology to a widely conserved chromatin regulator, pleckstrin homology domain‐interacting protein (PHIP). Deletion of the ARF ancillary domain, without compromising dimerization and B3 subdomains, impairs mutant complementation in Arabidopsis *arf5/mp* and *M. polymorpha arf1*, indicating the functional importance of the region. It was hypothesized that this ancillary domain may function as a histone reader, as it contains a hydrophobic cage associated with binding methylated histones, although individual mutations in this hydrophobic region ultimately had little effect. The ancillary domain plays an essential but unknown role distinct from methyl‐histone binding in ARF functionality (Hernández‐Garcia et al. 2024). More research is necessary to unravel the role the ancillary domain plays in ARF functional diversity.

Collectively, these findings underscore the complexity of auxin‐responsive gene regulation, highlighting the collaborative roles of AuxRE orientation, spacing, number, and coupling *cis*‐elements that integrate the influence of other transcription factors. The identification of biAuxREs and their functional validation reveals how ARFs can interact both cooperatively and competitively with diverse transcription factor families to regulate auxin‐responsive genes. Furthermore, recent insights into the ancillary domain have highlighted its integral but unclear role in ARF functionality. Ultimately, these factors likely all work together to modulate ARF affinity for and activity on distinct regulatory targets. The latest research on ARF DNA‐binding is reviewed in more detail by Rienstra et al. (2023).

### 3.2 The ARF Middle Region

While the N‐terminal region of ARFs mediates DNA binding, the middle region controls how bound genes are regulated. Unlike the inter‐class conservation observed in the DNA‐binding domain, the middle region of ARFs is the most highly divergent domain. ARF middle regions have been demonstrated to be sufficient for conferring activation or repression of transcription, delineated by ARF class: class A are activators, class B and C are repressors (Ulmasov et al. 1999b; Tiwari et al. 2003). Class A ARF middle regions are highly disordered, with some reported to contain a putative prion‐like domain, which has been implicated in regulating ARF availability (Powers et al. 2019). In contrast, Class B and C ARF middle regions are predicted to be less disordered and contain a B3 repression domain (BRD) or BRD‐like domain (Roosjen et al. 2018).

The leading hypothesis for the role of the middle region in ARF proteins is that it serves as a hub for interactions with other proteins (Cancé et al. 2022). These interactions appear to be separated largely by ARF class: chromatin remodelers and other transcriptional regulators (class A) or co‐repressors (class B/C). ARF5/MP (class A) has been shown to interact with SWI/SNF chromatin remodelers BRAHMA (BRM) and SPLAYED (SYD) via its middle region with bimolecular fluorescence complementation (BiFC), Co‐IP, and yeast‐2‐hybrid assays (Wu et al. 2015). The repression domain of class B ARFs contains a R/KLFG motif associated with the recruitment of the C‐terminal region of TPL and TPR co‐repressors (Ikeda and Ohme‐Takagi 2009; Lokerse and Weijers 2009; Choi et al. 2018). Class C ARFs contain a very similar sequence (VLFG), which is the basis of their association with repression. Interestingly, the middle region of ARF2 (class B) is unique in that it also contains an ethylene‐responsive element binding factor‐associated amphiphilic repression motif (EAR motif, characterized by an LxLxL sequence), which is associated with recruitment of the N‐terminal region of TPL/TPR and may have implications for co‐repressor binding affinity (Choi et al. 2018). In juxtaposition with ARF2, other EAR motif‐containing class B ARFs have the motif located within the C‐terminal PB1 domain (Choi et al. 2018). It remains unclear if this difference bears any functional relevance in regulating auxin‐responsive genes. Although interactions with TPL/TPR co‐repressors have been detected in a few class B ARFs, it is unclear whether this capability extends to other ARFs with R/KLFG or EAR motifs. For example, ARF19 (class A) is the only activator ARF that contains an EAR motif (Choi et al. 2018). Class B ARF middle regions may play additional roles beyond repression, as some have been shown to interact with BRM and SYD, typically associated with transcriptional activation (Efroni et al. 2013). ARF19 and class B ARFs might serve dual roles as both activators and repressors, depending on the co‐factors they recruit. Dual functionality to recruit distinct co‐factors could enable these ARFs to act as bimodal switches for auxin‐dependent gene regulation in different developmental contexts.

More recently, Morffy et al. (2024) developed a neural network trained to identify putative transcriptional activation domains. Consistent with their historical definition as ‘activator ARFs,’ all members of class A ARFs contained at least one predicted activation domain (Morffy et al. 2024). Class B and C ARFs generally lacked such a domain, but a few individuals (ARF1, ARF2, and ARF17) did score highly for containing potential activation domains. Comparing eleven flowering plant species, 97% of class‐A ARFs, 58% of class‐B ARFs, and 62% of class‐C ARFs have been predicted to have at least one activation domain (Morffy et al. 2024). However, in plants, the presence of repression domains, like those found in class B and C ARFs, often overrides the function of activation domains, likely maintaining their role as repressors *in vivo* (Hiratsu et al. 2003).

**FIGURE 4:**
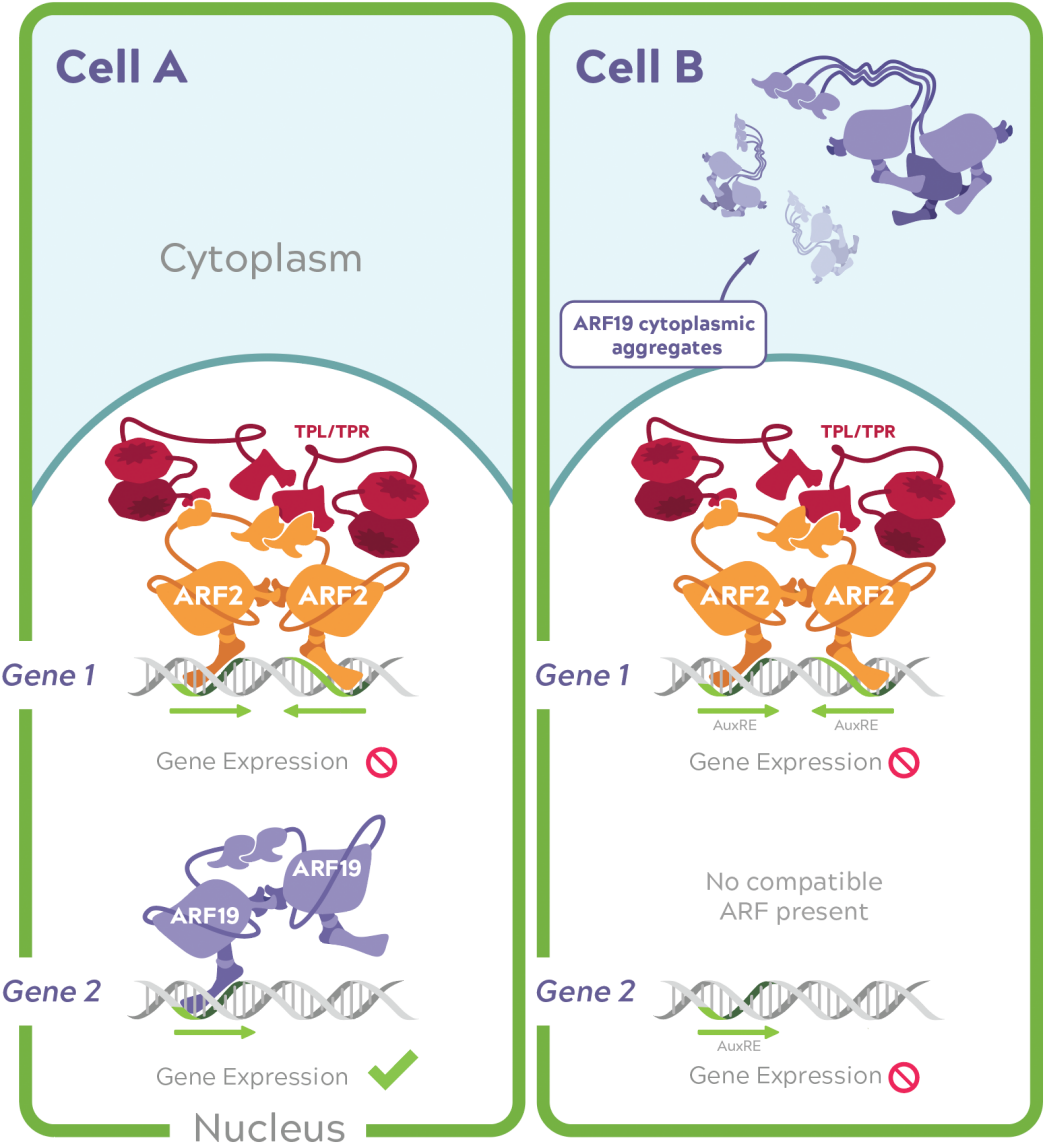
ARF‐mediated transcriptional specificity influenced by ARF condensate formation and AuxRE repeat number. In the nucleus of Cell A, ARF2 (a class B ARF; shown in orange) dimers bind to an AuxRE repeat sequence (green) and recruit TPL/TPR co‐repressors (red) to inhibit transcription of Gene 1, while ARF19 (a class A ARF; shown in purple) dimers bind a single AuxRE to activate Gene 2. In Cell B, ARF2 and TPL/TPR continue repressing Gene 1, which remains inactive in both cells. Meanwhile, ARF19 forms cytoplasmic aggregates, preventing its nuclear function. Without nuclear ARF19 and given that no other class A can bind a single AuxRE repeat, Gene 2 is not activated.

Another role of the ARF middle region may lie in its ability to regulate nucleocytoplasmic partitioning of certain ARFs (Figure 4). ARF7‐Venus and ARF19‐Venus fusions have been shown to form aggregate cytoplasmic structures (condensates) in mature root cells driven by the cooperative activity of the middle region and the PB1 domain (Powers et al. 2019). Mutations in either region disrupt the formation of condensates and lead to morphological defects and altered transcriptional responsiveness. Prion‐like domains, predominantly present in the class A ARFs, have been reported to self‐associate, promoting the formation of protein aggregates. ARF2, a class B ARF, which lacks a prion‐like region, does not form these condensates (Powers et al. 2019). The formation of ARF condensates likely regulates the availability of certain ARFs to control specific genes depending on the developmental stage or cellular context (illustrated in Figure 4). ARF condensates could also affect DNA‐binding or interactions with co‐factors to play a role in gene expression, as shown in other proteins with similar intrinsically disordered domains (Boija et al., 2018).

Recently, additional proteins have been implicated in regulating the stability of ARF condensates. MULTIPLE C2 DOMAIN AND TRANSMEMBRANE REGION PROTEINs (MCTPs) have been shown to interact with ARF7 and ARF19 to regulate their nucleocytoplasmic partitioning and root development. Specifically, MCTPs were shown to destabilize ARF7 and ARF19 condensates, promoting their nuclear localization (Xuan et al. 2024). This serves as another example of the role other proteins play in modulating auxin‐dependent gene regulation.

A final role of the middle region has been uniquely described for ARF3/ETTIN. The middle region of ARF3 contains an ARF3‐specific, C‐terminal subdomain that possesses an auxin‐binding site. This region remains conserved in ARF3 homologs in other species. Auxin binding to ARF3 has been shown to mediate its interactions with co‐repressors such as TPL/TPR and other transcription factors to regulate the activity of its target genes. Interactions of auxin with ARF3 results in its disassociation from complexes with co‐repressors, consequently derepressing the chromatin state (Simonini et al. 2017; Kuhn et al. 2020). ARF3 has also been shown to interact with chromatin remodelers BYD and SYD in yeast‐2‐hybrid assays, which may also play a role in ARF3‐driven chromatin changes to regulate gene activity (Efroni et al. 2013). ARF3 remains the only ARF shown to bind auxin, acting largely uniquely in plants as a DNA‐binding hormone receptor.

The ARF middle region plays a critical role in auxin signaling through its diverse roles in regulating how bound genes are regulated through protein–protein interactions, nucleocytoplasmic partitioning, and chromatin remodeling. The ARF class‐dependent functional variation in the middle region allows ARFs to recruit distinct co‐factors, acting as activators, repressors, or potentially serving dual roles, depending on developmental and environmental contexts. This emphasizes the importance and complexity of the ARF middle region in integrating signals to modulate gene expression in response to auxin.

### 3.3 The ARF PB1 Protein Interaction Domain

The C‐terminal PB1 domain, initially recognized as domains III/IV, present in both ARFs and Aux/IAAs, is critical to signaling specificity as it connects ARFs to auxin regulation by facilitating interactions between the two protein families (Ulmasov et al. 1997b; Tiwari et al. 2003). These interactions are essential for maintaining control of auxin signaling, as evidenced by the effects of PB1 domain deletion and mutagenesis. For example, expression of activator ARF proteins without a PB1 domain upregulates reporter‐gene activation in protoplasts, consistent with a loss of repression by Aux/IAAs (Tiwari et al. 2003; Wang et al. 2005; Gonzalez et al. 2021). Such ARF truncations also confer ‘high auxin’ phenotypes in plants, such as narrow, pointed lateral organs filled with parallel veins (Wang et al. 2005; Lau et al. 2011; Krogan et al. 2012a).

The characteristic structure of the PB1 domain is recognized by a Ubiquitin‐like β‐grasp fold of five β‐sheets and two α‐helices and is conserved in many distinct protein families across eukaryotes (Müller et al. 2006; Burroughs et al. 2007). The PB1 domain is typically composed of two oppositely charged faces (Burroughs et al. 2007; Ito et al. 2020). The N‐terminal face of the PB1 domain is positively charged with a conserved lysine residue, while the C‐terminal face is negatively charged with the conserved amino acid sequence (DX(D/E)XDXnD), known as the OPCA (OPR‐PC‐AID) motif. These charged faces permit homo‐ or heterotypic PB1 domain interactions in a head‐to‐tail manner (Burroughs et al. 2007; Korasick et al. 2015).

Mutation of the invariant lysine or the OPCA motif has been shown to be sufficient to disrupt homotypic interactions; however, these interactions are restored by mixing PB1 monomers with complementary mutations, confirming the directional nature of their interactions (Han et al. 2014; Korasick et al. 2014; Nanao et al. 2014; Dinesh et al. 2015). Truncated PB1 domains that lack one charged face, found in some non‐canonical class B and C ARFs (ARF10, 13–16, 20–21) and Aux/IAAs, can still interact with other PB1 domains to form dimers but not multimers (Guilfoyle 2015). There is some evidence that multimerization of certain Aux/IAAs is required for full repression of ARF activation (Korasick et al. 2015). Further research is required to determine whether sequence variation within individual PB1 domain faces influences ARF‐Aux/IAA interaction specificity and if pairing preferences have biological implications for transcriptional specificity.

Numerous studies have worked to elucidate the specificity of PB1‐mediated interactions involved in auxin signaling, predominantly via *in vitro* or heterologous protein–protein interaction assays (Dharmasiri et al. 2005; Vernoux et al. 2011; Causier et al. 2012; Wang et al. 2013; Piya et al. 2014). More recently, Luo et al. (2018) assembled a multi‐study, large‐scale interactome map of Aux/IAA proteins using data from affinity capture‐Western/MS, yeast‐2‐hybrid, reconstituted complexes, and protein‐fragment complementation assays to reveal the complex network of protein interactions at the core of the auxin signaling network. The analysis reported that the PB1 domain facilitates ARF and Aux/IAA interactions in accordance with an established framework, previously postulated by Vernoux et al. (2011): 1) Class A ARFs strongly interact with nearly all Aux/IAAs, 2) Aux/IAA proteins interact with themselves, and 3) Class B and C ARF repressors have very limited interactions with any other members of the network. Notably, heterotypic ARF‐Aux/IAA interactions exhibit approximately a 100‐fold greater affinity for each other over homotypic Aux/IAA interactions (Han et al. 2014). Piya et al. (2014) also performed serial dilutions of co‐transformed yeast cells in a yeast‐2‐hybrid assay to demonstrate that different ARF and Aux/IAA pairs exhibited distinct interaction strengths. The size of ARF and Aux/IAA protein families and the diversity of interaction affinities present opportunities for many combinatorial interactions to mediate auxin‐specific responses in various developmental and physiological contexts.

In contrast with class A ARFs, evidence for interactions of class B and C ARF‐PB1 domains with that of Aux/IAAs is limited. Only some class B ARFs (ARF1, 2, 4, 9, 12–14, 18, 20, 22) and C ARFs (ARF10 and 16) have been reported to interact with a small number of Aux/IAAs (Vernoux et al. 2011; Piya et al. 2014; Luo et al. 2018). Structural predictions suggest that differences in residues involved in H‐bonding and hydrophobic contacts likely contribute to the reduced affinity of repressor ARFs for Aux/IAAs (Parcy et al. 2016). A CrY2H‐seq assay reinforces that several class B/C repressor ARFs have distinct preferences for interactions with unique Aux/IAAs, which suggests that they likely serve specific functions, although this has yet to be demonstrated mechanistically (Wanamaker et al. 2017). Interestingly, ARF11 (class B) fails to interact with any Aux/IAAs despite maintaining the key residues in both the positive and negative PB1 domain faces required for interactions (Piya et al. 2014), which, if this is not an artifact of yeast two‐hybrid, suggests additional residues are likely involved conferring specificity for interactions with Aux/IAAs.

Recently, Hernández‐Garciá et al. (2024) uncovered more evidence for PB1‐mediated interaction specificity by exploring the shared ancestry of ARF and Aux/IAA PB1 domains with Related to ABI3 and VP1 (RAV) PB1 domains. A yeast‐2‐hybrid assay revealed that MpARFs exhibited strong homo‐ and heterotypic interactions with each other. In contrast, the MpRAV PB1 domain showed strong homotypic interactions and only interacted weakly with the PB1 domain of MpARF3 and MpIAA. Domain swaps of MpARF1 PB1 domain with that of MpRAV revealed that it was partially able to complement the mutant but disconnected the plants from auxin regulation. This indicated that the chimeric MpARF1 was still capable of oligomerization, which is required for its activity, but was not able to interact with MpIAA. Alternatively, swapping the MpARF1 PB1 domain with that of MpIAA was able to partially complement both the growth phenotype and auxin responsiveness (Hernández‐Garciá et al. 2024). This indicates that the RAV PB1 domains share partial functionality with ARF and Aux/IAA PB1 domains, but interaction affinities have likely diverged to favor specific PB1 interactions.

The involvement of additional elements beyond the PB1 domain has also been implicated in ARF‐Aux/IAA interaction affinities, as evidenced by inconsistent reports of multiple yeast‐2‐hybrid studies (Tiwari et al. 2003; Vernoux et al. 2011; Piya et al. 2014). These discrepancies appear to arise from the use of full‐length ARFs or isolated PB1 domains in yeast‐2‐hybrid experiments (Piya et al. 2014). For example, IAA17 interacts strongly with a full‐length ARF1, while the same interaction was not detected when only the ARF1 PB1 domain was used. Furthermore, ARF17, which lacks a PB1 domain, was reported to participate in a few weak interactions with select Aux/IAAs (Piya et al. 2014). These results point toward additional structural elements that modulate ARF‐Aux/IAA interaction beyond the PB1 domain.

The PB1 domain has also been implicated in interactions with proteins outside of the canonical nuclear auxin signaling pathway. For example, the ARF8 PB1 domain interacts with the transcription factor BIG PETALp (BPEp) in a BiFC assay in tobacco cells (Varaud et al. 2011). Additionally, the PB1 domains of ARF1, ARF7, IAA3/SHORT HYPOCOTYL 2 (SHY2), and IAA19/MASSUGU 2 (MSG2) interact with the MYB77 transcription factor, as shown by BiFC and pull‐down assays (Shin et al. 2007). Furthermore, several other PB1‐containing protein families are present in plants, such as nitrate signaling NIN‐LIKE PROTEIN transcription factors (NLP) (Hsin et al. 2021) and protein kinases/kinase‐derived proteins like CBS DOMAIN‐CONTAINING PROTEINS, and NEXT TO BRCA1 GENE 1 (NBR1) (Mutte and Weijers 2020). Further research is necessary to determine whether these proteins could interact with ARF or Aux/IAA PB1 domains and, if so, to study the biological significance of these interactions.

Additionally, some class B ARF (ARF12, 14–15, 20–22) PB1 domains contain a conserved TPL/TPR co‐repressor binding EAR motif (Choi et al. 2018). The functionality of this domain could enable TPL/TPR recruitment through both the ARF middle region and PB1 domain, but this has not been explored experimentally. Preferential recruitment of co‐repressors by certain class B ARFs combined with differing expression levels could elicit vastly different developmental responses, as evidenced by variation in TPL/TPR binding affinity by Aux/IAAs (Cho et al. 2024).

The PB1 domain appears to function as a diverse facilitator of interactions, from homo‐ and heterotypic interactions of ARF and Aux/IAAs to various other proteins outside the canonical nuclear auxin signaling pathway. While past research showcases the critical role of the PB1 domain in connecting ARFs to auxin regulation via interactions with Aux/IAAs, these studies almost invariably utilize *in vitro* or heterologous assays. In such assays, protein levels may far exceed those found in planta or may not be expressed in the same spatial or temporal context as their assumed partners. Also, the role of class B and C ARF interactions with Aux/IAAs remains unexplained. The complexity of ARF‐Aux/IAA partnerships emphasizes the need to clarify the role of these interactions in auxin signaling and whether pairing preferences play a role in transcriptional specificity. These interaction patterns seem to also vary by ARF class, but the biochemical basis for this specificity remains unclear. More work is necessary to determine the impact of these interactions on auxin signaling specificity.

## 4 AUX/IAA REPRESSORS

**FIGURE 5:**
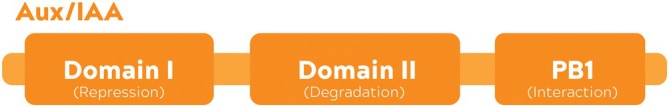
Schematic of the three functional domains found in canonical Aux/IAAs. N‐terminal Domain I: interacts with TPL/TPR co‐repressors to confer repression; Domain II: responsible for enabling auxin‐dependent interactions with TIR1/AFBs that facilitate Aux/IAA turnover; C‐terminal PB1 domain: confers interactions with ARF proteins and other Aux/IAAs.

**FIGURE 6:**
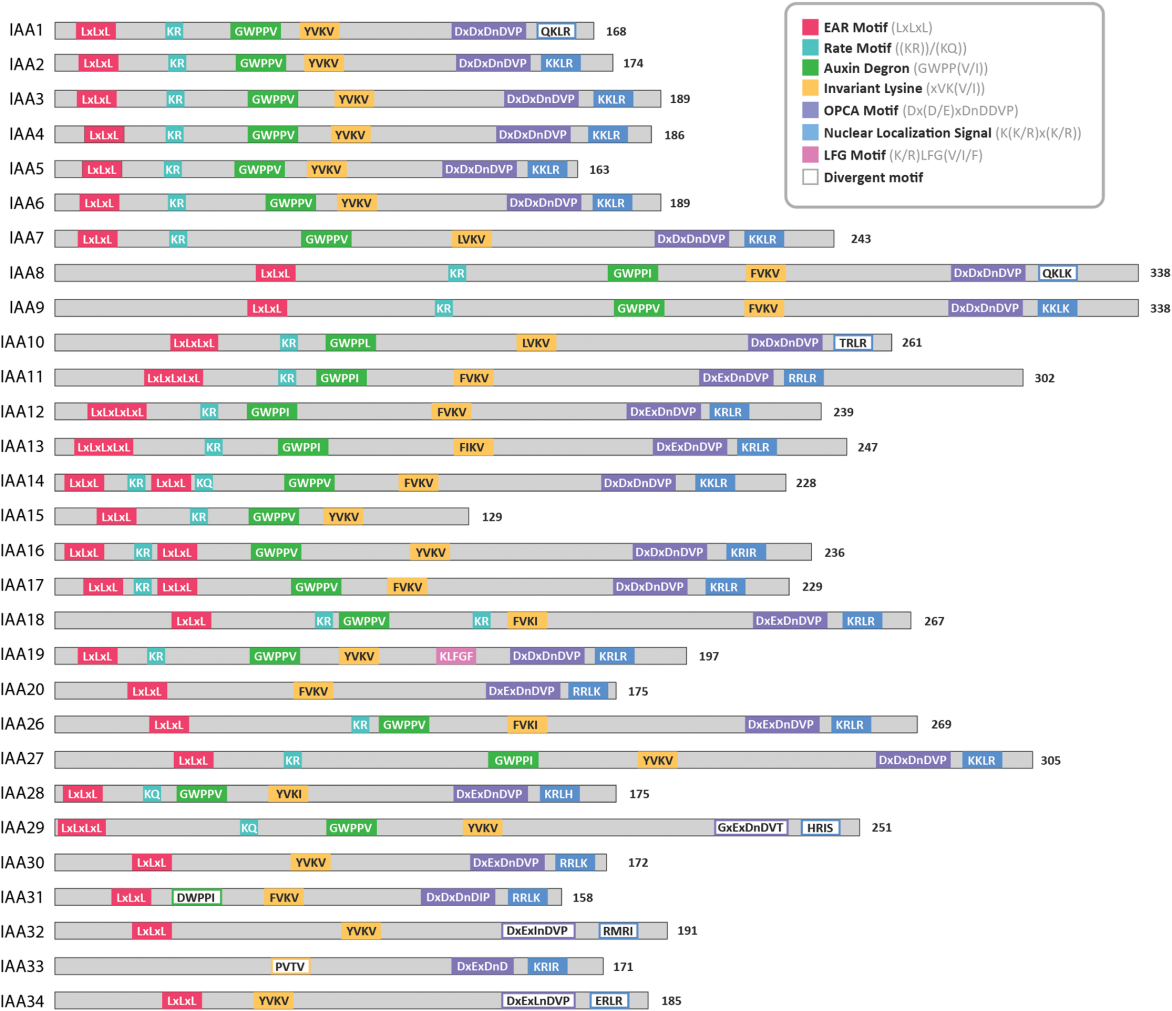
Schematic organization of conserved functional motifs of Aux/IAA proteins in Arabidopsis. The number of residues is indicated for each Aux/IAA. Sequences of key residues involved in the EAR repression motif (red), rate motif (teal), auxin degron (green), invariant lysine and directly adjacent residues (yellow), OPCA motif (purple), and monopartite nuclear localization signal (blue), and LFG repression motif (pink) are represented. Consensus sequences are indicated for each motif in the legend. Sufficiently divergent motifs are indicated by an unshaded box.

29 Aux/IAA proteins are encoded in the Arabidopsis genome; they act primarily to repress auxin signaling through three functional domains (Figure 5): the N‐terminal Domain I (also called the repression domain), responsible for recruiting transcriptional co‐repressors; Domain II (also known as the degron domain), which mediates auxin‐dependent interaction with TIR1/AFB proteins; and the C‐terminal PB1 domain (previously referred to as Domains III and IV), which enables interaction with ARFs and other Aux/IAAs (Figure 2, 5, 6). It is hypothesized that variations in these domains across the Aux/IAA family shape interaction specificity with ARFs and co‐repressors, while modulating Aux/IAA turnover to regulate specificity in transcriptional outputs mediated by auxin signaling.

While most Aux/IAAs have all three conserved domains, some lack at least one of these regions (Figure 6). Some are missing domains I and II, while others lack part or all of the PB1 domain (Luo et al. 2018). IAA20 and IAA31‐34 lack the degron domain entirely and IAA20 has been shown to be long‐lived and insensitive to auxin (Dreher et al. 2006); these Aux/IAAs may function predominantly as auxin‐independent heterodimerization partners (Luo et al. 2018). Additionally, domains I and II in half of Aux/IAAs are largely disordered, while these regions are more structured in other family members (Niemeyer et al. 2020). More work is necessary to reveal the developmental and transcriptional roles orchestrated through the diversity of the Aux/IAA family.

### 4.1 The Aux/IAA Transcriptional Repression Domain

The ability of Aux/IAA proteins to repress auxin‐induced gene expression lies with the recruitment of co‐repressor complexes through interaction with Domain I. Aux/IAA proteins were first confirmed as repressors by Ulmasov et al. (1997b) and Tiwari et al. (2001) when expressed in plant protoplast assays. Domain I of Aux/IAA proteins was shown to be required for repression and contains an EAR motif (LxLxL) (Figure 6), which functions to recruit TPL/TPR co‐repressors (Tiwari et al. 2001; Szemenyei et al. 2008). More than half of Aux/IAA proteins are reported to directly interact with four members of the TPL/TPR co‐repressor family (Luo et al. 2018) and deletion of Domain I results in a complete loss of repression (Tiwari et al. 2001). There is growing evidence that Aux/IAA sequence variations may have transcriptional implications depending on the developmental or environmental context and relative expression levels of individual Aux/IAA members. For example, Li et al. (2011) revealed that the same mutation in the EAR motif of different Aux/IAAs exhibits contrasting effects on the auxin response. Specifically, an alanine‐to‐leucine substitution in the EAR motif in IAA3/SHY2, IAA6/SHY1, or IAA19/MSG2 enhances auxin‐responsive gene expression, whereas the same substitution in IAA12/BDL or IAA17/AXR3 represses auxin‐responsive genes (Li et al., 2011). These findings highlight structural differences in Domain I of Aux/IAA proteins, where some may have stronger or more complex repressive regions than others. These differences may have transcriptional implications depending on which Aux/IAAs are expressed in a particular developmental context.

More recently, Cho et al. (2024) identified differences in key residues within the EAR motif of some Aux/IAAs that confer differing affinities for TPL/TPR proteins. It was revealed that replacement of a single amino acid residue (X_1_ of Lx_1_Lx_2_L) in the TPL/TPR‐binding motif elicited vastly different responses related to auxin‐mediated root‐hair growth. Specifically, IAA7/AXR2 was only associated with repression of root‐hair growth, while IAA3/SHY2 served a bimodal role to either promote or inhibit root‐hair growth, depending on its relative expression levels. The authors proposed a model in which Aux/IAAs differ in co‐repressor‐binding affinities and, in turn, compete to act as a dose‐dependent transcriptional switch (Cho et al. 2024). They show that IAA3/SHY2 may interact relatively weakly with TPL/TPR co‐repressors. At moderate expression levels, IAA3/SHY2 could competitively bind to ARF proteins, reducing transcriptional repression by other, strong TPL/TPR‐binding Aux/IAAs (illustrated in Figure 6). These findings were supported by several lines of experimental evidence, such as auxin‐induced root‐hair growth inhibition studies and Co‐IP. Interestingly, several other Aux/IAAs contain EAR motifs identical to IAA3/SHY2, which raises the question if they confer similar bimodal responses in their respective expression domains.

Variation in Domain I underscores the complexity of Aux/IAA‐mediated repression in auxin signaling. Differing EAR motif configurations and relative expression levels highlight how Aux/IAAs function as nuanced regulators, suggesting an inherent specificity for Aux/IAAs to drive distinct transcriptional outputs. More research is necessary to uncover how these structural variations contribute to the precise control of auxin signaling specificity across diverse developmental and environmental contexts.

### 4.2 The Aux/IAA Degradation Domain

The interaction between Aux/IAAs and TIR1/AFB receptors is fundamental to auxin‐induced degradation of the repressors (Calderon‐Villalobos et al. 2010). This interaction occurs within Domain II, also known as the degradation domain. Aux/IAA proteins have a short half‐life relative to other proteins (Worley et al. 2000; Dreher et al. 2006) and mutations in Domain II often result in reduced turnover and decreased auxin responsiveness (Ramos et al. 2001; Zenser et al. 2003). Aux/IAAs that lack Domain II (e.g., IAA20) are auxin insensitive and exhibit significantly slower rates of turnover (Dreher et al. 2006). Sequence alignments and truncation studies have uncovered a degron core “GWPP(V/I)” within a largely conserved 13 amino acid sequence in Domain II, which is required for auxin‐induced degradation (Worley et al. 2000; Gray et al. 2001; Ramos et al. 2001; Zenser et al. 2001). Aux/IAAs and TIR1/AFBs form an auxin‐coreceptor system where auxin fills a pocket in TIR1/AFB, just beneath the Aux/IAA degron region (Tan et al. 2007). The auxin‐dependent binding kinetics of the receptors are predominantly determined by the specific Aux/IAA involved. Consequently, distinct Aux/IAA and TIR1/AFB combinations have unique auxin sensing properties (Calderón Villalobos et al. 2012). Mutations in Domain II alter Aux/IAA affinity for interactions with the TIR1/AFB, turnover rates, and elicit abnormal plant phenotypes (Dreher et al. 2006; Moss et al. 2015). For example, the turnover rate of IAA14/SLR is intimately tied to lateral root development and mutants with reduced turnover formed less lateral roots (Guseman et al. 2015).

In addition to the degron motif, additional residues have also been implicated in contributing to Aux/IAA turnover. E3 ubiquitin ligases such as SCF^TIR1/AFB^ ubiquitylate one or more specific lysine residues in their targets (Mattiroli & Sixma 2014). Between Domain I and II is a conserved lysine‐arginine (KR) motif that has been identified as a “rate motif” that aids in tuning Aux/IAA turnover (Moss et al. 2015). These rate motifs belong to flexible, lysine‐enriched disordered regions flanking the core degron motif. Aux/IAAs with a KQ motif instead of KR have reduced auxin sensitivity (Dreher et al. 2006; Calderon Villalobos et al. 2012; Moss et al. 2015). Although they do not appear to affect Aux/IAA interaction affinities for TIR1/AFBs, mutating the rate motif significantly decreases protein turnover. Plants expressing Aux/IAA proteins without this rate motif have auxin‐insensitive phenotypes, like those conferred by degron mutations (Moss et al. 2015). Moreover, an additional rate motif has been reported on the flanking C‐terminal sequence to the degron region. This region is enriched in polar residues and its deletion results in reduced affinity for interactions with TIR1/AFB proteins and decreased turnover (Moss et al. 2015). Furthermore, flexible regions containing conserved lysine residues in the degron‐flanking regions have been observed to confer differences in ubiquitylation, as shown for IAA6/SHY1 and IAA19/MSG2 (Winkler et al. 2017). Differences in the rate motifs and flexible regions between Aux/IAAs alter their ubiquitylation and degradation kinetics potentially to elicit specific auxin responses.

Recently, an investigation of the intrinsically disordered nature of Aux/IAA domains I and II revealed that this region in IAA17/AXR3 has a propensity to adopt a few specific conformations. Using nuclear magnetic resonance, circular dichroism, and molecular dynamics simulations, the work by Ramans‐Harborough et al. (2023) demonstrated that this region of IAA17/AXR3 is nominally disordered. While the N‐terminus of over half of Aux/IAAs are generally considered to be disordered, IAA17/AXR3 displayed a tendency towards the formation of two distinct conformations. Notably, they identified a crucial *cis*–*trans* isomerization at the conserved W–P region within the degron core, with an unusually high 1:1 ratio of *cis* to *trans* isomers. These two conformational arrangements form different auxin coreceptor complexes, which may influence Aux/IAA turnover. Additionally, these two conformations appeared to influence the deployment of the PB1 domains, which likely influences many biological interactions (Ramans‐Harborough et al. 2023). Together, these findings provide new insight into how structural diversity within Aux/IAAs could regulate their turnover and interactions to facilitate distinct transcriptional responses.

The complexity of Aux/IAA turnover kinetics emphasizes its critical role in fine‐tuning auxin signaling specificity. These findings show how variations in degron motifs, rate motifs, and flexible regions of Aux/IAAs modulate protein turnover to tune signaling dynamics and regulate context‐specific developmental and physiological processes.

### 4.3 The Aux/IAA PB1 Protein Interaction Domain

Aux/IAA proteins interact with ARF proteins via their shared PB1 domains to confer transcriptional repression. Korasick et al. (2014) suggested that some Aux/IAA proteins multimerize on ARF PB1 domains to fully repress ARF activity. To test this, they deployed stabilized (gain‐of‐function) IAA16 with various PB1 mutations in Arabidopsis. Expression of the stabilized mutant *iaa16‐1* had a deleterious impact on plant growth, while mutations to the invariant lysine or OPCA motif of the PB1 domain (*iaa16‐1* ^*K112A*^, *iaa16‐1* ^*opca*^) resulted in wild‐type phenotypes. These PB1 domain mutants still retained one of the two interaction faces and thus were still capable of dimerization with ARF proteins. These results suggest that multimerization may be required for IAA16 to confer repressive activity (Korasick et al. 2014) and are backed up by similar experiments in rice with OsIAA23 (Ni et al. 2014). Expression of non‐multimerizing IAA17/AXR3 and IAA19/MSG2 in protoplasts also reduced the repressive activity (Nanao et al. 2014). This has also been observed in *Physcomitrella patens*, where a multimerization of IAA1a is required for full repression (Tao and Estelle 2018). Multimerization of certain Aux/IAAs has been reported to enhance TPL/TPR recruitment as evidenced by structural analysis, which suggested that TPL/TPR binding affinity increases with multimerized Aux/IAA complexes (Ke et al. 2015). In contrast, an engineered non‐multimerizing IAA14/SLR isoform is still able to efficiently repress auxin response using a recapitulated auxin response circuit in yeast (Pierre‐Jerome et al. 2016).

A recent study has also implicated the PB1 domain in auxin‐dependent interactions with TIR1. Niemeyer et al. (2020) revealed that IAA12/BDL and IAA7/AXR2 complex with auxin and TIR1 with different affinities due to differences in their degron tail and PB1 domain. In a yeast‐2‐hybrid assay, swapping the degron tail or PB1 domain between IAA7/AXR2 and IAA12/BDL affected interactions with TIR1, with the IAA7/AXR2 PB1 domain playing a key role in auxin‐dependent binding. Follow‐up *in vitro* radioligand assays confirmed that altering IAA7/AXR2's degron tail or PB1 domain reduced binding affinity, whereas IAA12/BDL variants exhibited minimal changes, suggesting an additive effect of the IAA7/AXR2 PB1 domain in stabilizing interactions with TIR1 (Niemeyer et al. 2020).

Ultimately, the functional diversity of Aux/IAAs has begun to clarify how individual members may contribute to distinct auxin‐mediated transcriptional responses. Differences in TPL/TPR binding affinity through Domain I could allow certain Aux/IAAs to act as activators or repressors depending on their relative availability. Variations in Domain II regulate Aux/IAA availability by modulating their turnover rates. Finally, the PB1 domain plays multiple roles in regulating interaction affinities with ARFs, TIR1/AFBs, and facilitates Aux/IAA multimerization, which is required for repression in some instances. These studies underscore the functional diversity of Aux/IAA proteins and the nuanced ways such differences may tune auxin signaling. Further work is necessary to elucidate the structural properties that contribute to these differences and uncover their impact on auxin signaling specificity.

## 5 THE TIR1/AFB RECEPTORS

TIR1/AFB proteins facilitate auxin perception as a part of the SCF^TIR1/AFB^ E3 ubiquitin‐ligase complex, acting as auxin receptors that mediate the degradation of Aux/IAA proteins. Crystal structures have demonstrated that auxin binds a hydrophobic region in TIR1/AFB proteins, stabilizing their interaction with Aux/IAA proteins while other members of the SCF complex mediate the polyubiquitylation that leads to subsequent degradation of Aux/IAA proteins (Tan et al. 2007).

There are six *TIR1/AFB* genes encoded in the Arabidopsis genome that exist as three pairs of paralogs, which likely confer functional redundancy while allowing novel functions to evolve (Parry et al. 2009; Shimizu‐Mitao and Kakimoto 2014). Reverse‐genetic studies have uncovered additional redundancies between TIR1, AFB2, and AFB3, and separately, between AFB4 and AFB5 (Dharmasiri et al. 2005; Prigge et al. 2016). A single wild‐type allele of TIR1 or AFB2 is sufficient to support plant development after loss of function mutations in all six TIR1/AFB genes (Prigge et al. 2020). However, AFB1 and AFB2 fail to rescue the *tir1* mutant even under the *TIR1* promoter (Parry et al. 2009). Furthermore, AFB1 has been implicated in the fast auxin response (Prigge et al. 2020) in contrast with TIR1 (Qi et al. 2022). Notably, AFB1 recently diverged from TIR1 and is specific to Brassicales, suggesting a lineage‐specific specialization. Additionally, more divergent AFBs are found outside Brassicales, but these were lost specifically in this lineage (Prigge et al. 2020). The lineage‐specific differences of TIR1/AFB family members point toward specialized functional roles among TIR1/AFB family members that likely contribute to the fine‐tuning of auxin signaling specificity.

TIR1/AFB proteins participate in the formation of the SCF complex and auxin‐mediated degradation of Aux/IAA proteins via three functional domains (Figure 8): an N‐terminal F‐box domain, known for regulating protein turnover (Jain et al. 2007); a leucine‐rich LRR region broadly associated with protein–protein interactions (Martin et al. 2020; Prigge et al. 2020); and a recently identified adenylate cyclase motif in the C‐terminus (Qi et al. 2022). There are also many other lesser‐known motifs that are conserved in some or all TIR1/AFB members (Du et al. 2022). How these differences contribute to the distinct functional roles of TIR1/AFB proteins or if they generate unique transcriptional outputs is not yet fully understood. These motifs could conceivably confer differences in Aux/IAA turnover by affecting binding affinity or instilling preferences for distinct auxin species.

### 5.1 The TIR1/AFB Auxin Binding Pocket

TIR1/AFB proteins interact with and facilitate the degradation of Aux/IAAs in an auxin‐dependent manner (Dharmasiri et al. 2005; Tan et al. 2007). Tan et al. (2007) demonstrated, using an electrophoretic mobility shift assay, that auxin enables the TIR1–Aux/IAA interaction, which is undetectable in the absence of auxin. Furthermore, crystal structures of TIR1 and IAA7/AXR2 revealed a single auxin binding pocket within the LRR domain (Tan et al. 2007). These structures suggested that the binding pocket can accommodate various auxin species with the Aux/IAA protein docking “on top” with auxin acting as a “molecular glue” facilitating the interaction of the two proteins.

The auxin‐binding pockets in TIR1/AFB family members have slight differences, which may indicate preferences for distinct auxin species. This is supported by Arabidopsis mutants, which have revealed that different TIR1/AFB family members are responsible for the action of specific synthetic auxins used as herbicides (Grossmann et al. 1996). While AFB5 and TIR1 have similar preferences for Aux/IAA degron motifs and similarly interacted with most auxin compounds (aside from the higher affinity of picloram for AFB5; Calderón‐Villalobos et al. 2012), accelerated disassociation rates were observed with AFB5 (Lee et al. 2014). Moreover, *tir1* mutants are resistant to 2,4‐D while the closely related *afb1* mutants are not (Gleason et al. 2011).

Indole‐3‐acetic acid (IAA) is the dominant form of auxin across plant and algal species (reviewed in Lau et al. 2009). Other endogenous auxin species, indole‐3‐butyric acid, phenylacetic acid, and 4‐chloroindole‐3‐acetic acid are not as well studied (reviewed in Simon and Petrášek 2011). The evidence of distinct preferences by TIR1/AFB members for synthetic auxins suggests that individual receptors may also exhibit preferential interactions for endogenous auxins. Whether this bears any functional significance on signaling specificity, such as contributing to distinct Aux/IAA interaction affinities, requires additional investigation.

### 5.2 TIR1/AFB Interactions with Aux/IAAs

TIR1/AFB proteins have been shown to form preferential interactions with specific Aux/IAA family members. Calderón‐Villalobos et al. (2012) demonstrated via yeast‐2‐hybrid and immunoblot assays that Aux/IAA proteins bind to TIR1/AFB proteins with different affinities. For example, IAA3/SHY2 interacts with AFB1 at 0.1 μM IAA, but not TIR1, AFB2, or AFB5. However, at 100 μM, IAA3/SHY2 now binds TIR1 and AFB2 but still fails to bind AFB5. Interestingly, IAA7/AXR2 could interact with TIR1/AFBs in the absence of auxin and IAA31, which contains a non‐canonical degron, “DWPPI,” interacted only weakly (Calderon Villalobos et al. 2012). Additionally, certain Aux/IAA proteins, (IAA3/SHY2, IAA5, IAA7/AXR2, and IAA8) were reported to interact with TIR1/AFBs with higher affinities than others (IAA12/BDL, IAA28, and IAA31). Since all the tested Aux/IAAs contained the degron motif, these results indicate that additional residues determine Aux/IAA affinity for TIR1/AFBs. Furthermore, saturation/homologous competitive auxin (IAA) binding assays showed that Aux/IAA proteins influence TIR1/AFB affinity for auxin. These assays have also suggested that Domains I and II of Aux/IAA proteins may be required for the receptor to have full auxin‐sensing capabilities (Calderon Villalobos et al. 2012). Interestingly, interactome data hosted on BioGRID v4.4.239 (https://thebiogrid.org) only contains reports of ten Aux/IAAs as having direct interactions with five of the six TIR1/AFB proteins despite most Aux/IAAs possessing the core degron motif. This further supports the model of additional elements outside the degron motif contributing to the receptor affinity for Aux/IAAs, confirmed by studies in yeast (Moss et al. 2015; Gilkerson et al. 2015).

Adding another layer of complexity, there is evidence of proteins that antagonize Aux/IAA interactions with TIR1/AFBs, inhibiting their turnover. For example, RGA‐LIKE3 (RGL3) competitively interacts with IAA17/AXR3 to protect it from TIR1‐mediated degradation (Shi et al. 2017). Moreover, Dezfulian et al. (2016) demonstrated that the homo‐oligomerization of TIR1 in Arabidopsis served as another mechanism for regulating Aux/IAA protein turnover. A yeast‐2‐hyrbid assay and Co‐IP using *Nicotiana benthamiana* and Arabidopsis were used to verify TIR1 homotypic interactions. TIR1 homo‐oligomerization mutants failed to rescue dampened auxin‐responsive phenotypes, which suggests these interactions are essential for mediating Aux/IAA turnover (Dezfulian et al. 2016). Homo‐oligomerization of TIR1/AFBs and additional interactions contributing to Aux/IAA turnover remain relatively unexplored. Determining whether these interactions bear biological significance will require further investigation.

### 5.3 TIR1/AFB Subcellular Localization

Recent work has implicated cellular localization of TIR1/AFBs in their function during auxin‐induced degradation of Aux/IAAs. TIR1 is predominantly localized to the nucleus, while AFB1 is localized to the cytosol and is associated with fast auxin responses (Prigge et al. 2020). It has recently been shown that the nuclear import of TIR1 and AFB2 is regulated by oxidative modifications. Through liquid chromatography–tandem mass spectrometry and parallel reaction monitoring, Lu et al. (2024) demonstrated that the FERONIA‐reactive oxygen species (ROS) signaling pathway mediates cysteine oxidative modifications on TIR1 and AFB2. Stimulated by auxin, these oxidative events, facilitated by the FERONIA‐ROS pathway, orchestrate the nuclear transport of these TIR1 and AFB2 (Lu et al. 2024).

Recently, Chen et al. (2023) tested whether receptor localization is important for their function in transcriptional regulation. They found that targeting TIR1 to the cytosol in a *tir1* mutant Arabidopsis line was able to completely restore auxin sensitivity. This could be explained by residual TIR1 proteins still present in the nucleus or indicates that a cytosolic TIR1 can still sufficiently degrade Aux/IAA proteins (Chen et al. 2023). Mistargeting TIR1 to the cytosol and AFB1 to the nucleus in *afb1* background plants, however, could not complement the mutant phenotype. This provides further evidence that TIR1 and AFB1 play unique roles beyond their subcellular localization. To test this further, Chen et al. (2023) employed a minimal auxin circuit in yeast. There, they found that TIR1, but not AFB1, could facilitate the auxin‐induced transcriptional response, regardless of its subcellular localization (Chen et al. 2023). This might be due to AFB1 not interacting with CULLIN 1 (CUL1), a key component of the SCF complex, indicating that CUL1 may not be required for AFB1‐mediated rapid auxin response.

Furthermore, Dubey et al. (2023) revealed, through domain swaps in AFB1, that beyond the F‐box domain, the N‐terminal region supports auxin binding and is essential for fast auxin response. Substitution of the AFB1 N‐terminal region with that of TIR1 destabilizes AFB1's characteristic cytoplasmic localization and auxin‐mediated calcium influx, both required for regulation of rapid root growth inhibition. It was also reported that AFB1 negatively regulates lateral root development and auxin‐responsive gene expression, implying that it acts as an inhibitor in auxin signaling (Dubey et al. 2023).

Together, these findings affirm that TIR1/AFBs play distinct roles in auxin signaling, with TIR1 mediating slow auxin responses predominantly from the nucleus and AFB1 mediating rapid responses in the cytosol. These functional differences are not simply due to the differential subcellular localization of these auxin receptors, pointing to other factors that contribute to the distinct roles played by each of these receptors in auxin regulation. How these differences regulated distinct developmental and transcriptional responses requires further investigation.

### 5.4 Adenylate Cyclase Activity of TIR1/AFBs

In a quest to characterize the mechanism that underlies rapid auxin‐dependent root responses, Qi et al. (2022; summarized by Wong et al. 2022) uncovered that TIR1/AFBs have adenylate cyclase (AC) activity, suggesting that the receptors produce cAMP. Sequence analysis revealed a conserved AC amino acid motif in the C‐terminal region of TIR1/AFBs in Arabidopsis and in *P. patens*, suggesting an ancient evolutionary origin. The motif confers similar catalytic activity as other adenylate cyclases found in plants that contain the same motif. Structural analysis of the TIR1‐auxin‐Aux/IAA co‐receptor complex revealed that the auxin‐binding pocket is positioned close to the AC motif and further modeling suggested that cAMP production is enhanced by auxin‐dependent co‐receptor complex formation. Measuring cAMP levels in Arabidopsis mutants after auxin treatment confirmed these findings. Despite the efforts to uncover the molecular mechanism behind rapid auxin‐dependent root responses, the authors found that AC activity in TIR1/AFB is not required for these rapid responses. Mutations disrupting AC activity in TIR1 did not affect auxin‐induced root growth inhibition, nor did they alter cytosolic Ca^2+^ levels or apoplast alkalinization, both hallmarks of the rapid auxin‐induced root growth inhibition through TIR1 (Fendrych et al. 2018; Qi et al. 2022). Instead, AC activity was primarily linked to longer‐term transcriptional auxin responses, such as the activation of genes like *GRETCHEN HAGEN 3.3* (*GH3.3*), *GH3.5*, *IAA5*, *IAA19/MSG2*, and *LBD29*, as demonstrated by qPCR (Qi et al. 2022).

These findings add to the current model of auxin signaling by demonstrating that TIR1/AFBs also function as adenylate cyclases, connecting cAMP to auxin transcriptional response. While AC activity does not appear to be involved in rapid root growth inhibition, it plays a crucial role in auxin‐mediated transcriptional responses.

## 6 ADDITIONAL LAYERS OF REGULATION

Beyond the core components of the nuclear auxin signaling pathway, many additional factors likely contribute to auxin signaling specificity. These include transient versus sustained auxin signaling, co‐expression patterns, post‐translational modifications, regulatory RNAs, and crosstalk with other pathways. While evidence for their contributions is growing, their precise roles in auxin‐regulated transcription remain incompletely understood. The following sections briefly discuss these factors and their potential to further tune auxin signaling specificity.

### 6.1 Transient versus Sustained Auxin Signaling

The focus of the role auxin signaling plays in development has largely been centered around the spatial specificity of auxin signaling machinery to clarify how different cells respond to auxin. Both transcriptional and non‐transcriptional auxin responses occur on the seconds to minutes or hour time scales, requiring complex quantifications over time. As a result, many studies have neglected to explore the possibility of temporal contributions to auxin signaling to regulate plant development, except for some rapid responses such as gravitropism. Recent work has indicated that auxin‐dependent transcriptional regulation may not always be a fast process (reviewed in more detail in Caumon and Vernoux 2023).

Lateral root priming exemplifies how the temporality of auxin signaling regulates plant development. Priming is the formation of pre‐branching sites composed of groups of pericycle cells from which the lateral root will ultimately develop (Torres‐Martínez et al. 2022). These sites establish themselves with constant spacing and are characterized by cyclical expression of thousands of auxin‐responsive genes in the oscillation zone (Moreno‐Risueno et al. 2010). Two competing models explain this rhythmic patterning: the root clock model, where gene oscillations are cell‐autonomous and regulated by an ARF7‐IAA18 negative feedback loop (Perianez‐Rodriguez et al. 2021); and the reflux‐and‐growth model, where auxin loading and cell growth generate oscillatory auxin concentrations and subsequent activation of auxin‐responsive genes (van den Berg et al. 2021). The second model follows a two‐step mechanism, which first facilitates the initiation of the pre‐branching site by transient increases in auxin concentrations in the oscillation zone. This is then followed by a more constant elevation in auxin signaling to continue site establishment (Santos Teixeira et al. 2022). Both models illustrate how the temporality of nuclear auxin signaling from either integration into a broader gene oscillation network or as an intrinsic feature of the pathway is critical for rhythmic organ patterning. Additionally, studies on IAA14/SLR degradation show that protein turnover influences lateral root density and timing, highlighting the role of auxin signaling duration in developmental coordination (Guseman et al. 2015).

Auxin distribution in the root also simultaneously regulates root meristem zonation (Sabatini et al. 1999; Ding and Friml 2010) and gravitropism, yet these processes require different signaling kinetics. In the root meristem, a proximo‐distal auxin gradient maintains the stem cell pool (Sabatini et al. 1999) while triggering differentiation (Di Mambro et al. 2017) mediated by PLETHORA (PLT) 1 and 2 proteins (Galinha et al. 2007; Ding and Friml 2010). During gravitropism, auxin rapidly accumulates on the lower root side to drive bending (Su et al. 2017). Despite this redistribution, meristem patterning remains stable due to differences in auxin response timing: PLT2 activation is slow, requiring days to form a protein gradient along the proximo‐distal axis, whereas gravitropic auxin signaling is rapid, occurring within minutes to hours (Mähönen et al. 2014). This segregation of fast and slow auxin responses allows auxin to regulate multiple developmental processes simultaneously without disruption.

Organ initiation in the shoot apical meristem (SAM) serves as another example of the role timing plays in auxin signaling. In the SAM, organ initiation follows a spatio‐temporal pattern dictated by auxin accumulation, establishing phyllotaxis, the arrangement of organs around the stem. Recent high‐resolution imaging revealed that several auxin maxima are present simultaneously in the zone of organogenesis in the SAM, even in locations where no organ initiation or auxin response is detected (Galvan‐Ampudia et al. 2020). This study also uncovered that auxin concentration and transcriptional response are not always correlated. This indicates that higher cellular concentrations of auxin do not necessarily immediately result in higher transcriptional response. Exogenous applications of auxin revealed a requirement for prolonged auxin exposure for cells in the SAM to start activating a transcriptional response associated with organ initiation (Galvan‐Ampudia et al. 2020). While the molecular mechanism for this remains unknown, this suggests that auxin acts as a temporal cue, with cells integrating auxin signals over time rather than responding immediately. This challenges the conventional view that auxin‐induced transcription occurs within minutes and highlights how temporal auxin integration ensures robust, iterative organ patterning at the SAM, like rhythmic patterning in the root. In this context, the temporal integration of the nuclear auxin pathway may drive organ formation in the SAM based on auxin exposure history rather than immediate concentration.

### 6.2 Co‐expression Patterns

Co‐expression patterns have been shown to modulate the availability of signaling machinery, regulating available players to create tissue‐specific interaction networks. Piya et al. (2014) integrated co‐expression profiles with protein–protein interaction data to reveal that 70% of ARF‐Aux/IAA interacting pairs exhibit strong gene co‐expression in at least one tissue or organ. For example, ARF7‐IAA19/MSG2 were found to interact and be co‐expressed in the hypocotyl, while ARF7/19‐IAA14/SLR and ARF7/19‐IAA28 “co‐function” in roots. Notably, ARF4‐8 and ARF19, which interact with nearly all Aux/IAA proteins, displayed broad co‐expression patterns with Aux/IAA genes, implicating these proteins as central hubs within the co‐expression network (Piya et al. 2014). These findings underscore the importance of co‐expression patterns and highlight the functional significance of unique ARF‐Aux/IAA pairs shaping tissue‐specific gene expression.

### 6.3 Regulatory RNAs and Alternative Splicing

Post‐transcriptional regulation at the RNA level has also been implicated in tuning auxin signaling. Modulation by microRNAs and alternative splicing of nuclear auxin pathway machinery make up the core regulators at this level. mRNA turnover for some repressor ARFs and TIR1/AFBs have been shown to be regulated by microRNAs (Parry et al. 2009; Huang et al. 2022). For example, recent work has revealed additional evidence of ARFs being regulated by microRNAs, in which ARF17 is targeted by miR160 to tune ovule development (Huang et al. 2022). These findings suggest that mRNA regulation of core auxin signaling machinery is important to developmental responses to auxin.

Furthermore, certain splice variants of ARF proteins have been shown to lack key domains, which potentially alters their function. For example, alternative splicing of ARF5/MP in the ovule leads to an isoform lacking a PB1 domain that functions independently of Aux/IAA regulation (Cucinotta et al. 2021). Moreover, a recent paper revealed that the turnover of particular ARF5/MP isoforms are differentially regulated depending on the auxin level in the root. The ARF5/MP isoform MP11ir lacks the PB1 domain, allowing it to activate gene expression, while the full‐length form interacts with IAA12/BDL, leading to ARF5/MP proteasomal degradation (Cavalleri et al. 2024). However, the biological significance and frequency of alternative splicing of other members of the nuclear auxin pathway components requires additional investigation.

### 6.4 Post‐translational Modifications

Post‐translational modifications have also been shown to add additional layers of regulation to auxin signaling. For instance, S‐nitrosylation of TIR1 enhances its affinity for interaction with Aux/IAAs, increasing their degradation (Terrile et al. 2012), while *cis‐trans* isomerization of proline residues in Aux/IAAs has been shown to affect auxin sensitivity (Dharmasiri et al. 2003; Jing et al. 2015). Moreover, SUMOylation of Aux/IAA proteins can also reduce their turnover, while it has been shown to inactivate some ARF proteins (Orosa‐Puente et al. 2018; Zhang et al. 2023). Aux/IAA proteins have also been reported to be phosphorylated by phytochrome A *in vitro*, and phosphorylation of ARFs has been reported as well (Colón‐Carmona et al. 2000; Cho et al. 2014). BIN2 kinase phosphorylates ARF7 and ARF19, enhancing their transcriptional activity to promote lateral root organogenesis, whereas BIN2 phosphorylation of ARF2 reduces its DNA‐binding ability and repressor activity (Cho et al. 2014). In addition to Aux/IAAs, ubiquitylation may also regulate ARF turnover (Salmon et al. 2008; Vert et al. 2008). Although many of these modifications require additional investigation *in vivo* to determine their biological significance, they highlight the dynamic regulatory potential of post‐translational modifications in modulating auxin signaling.

### 6.5 Hormone Signaling Crosstalk

Auxin does not act in isolation to mediate diverse developmental responses; its signaling machinery has been shown to interact extensively with other hormonal pathways, e.g., cytokinin, brassinosteroids, gibberellin, abscisic acid, jasmonic acid, and ethylene (Wang et al. 2007; Zemlyanskaya et al. 2018; Mallick et al. 2022). For example, the transcription factor BRASSINAZOLE RESISTANT 1 from the brassinosteroid pathway and the gibberellin signaling protein REPRESSOR of GA both interact with the middle region of ARF6 to regulate auxin‐dependent gene expression (Oh et al. 2014). These findings demonstrate the complexity of hormone signaling crosstalk and its involvement in regulating auxin signaling specificity.

## 7 WHERE DO WE GO FROM HERE?

While substantial progress has been made in understanding the mechanisms that underlie auxin signaling specificity, unanswered questions remain. For example, do ARFs heterodimerize to regulate a variety of differently spaced and oriented AuxRE pairs? ARF heterodimerization could help further tune transcriptional outputs in response to auxin, allowing unique ARF combinations to recognize different AuxRE pairs. Heterodimerization may enable ARF binding to AuxREs with a variety of different configurations and affinities, also recruiting distinct co‐factors and transcriptional machinery.

In addition, the ARF middle region remains relatively unexplored. For instance, do ARFs that possess both activation and repression domains serve dual roles *in vivo*? Morffy et al. (2024) investigated the evolution of activation domains in class A ARFs within flowering plants. While the activation domains within these subclades showed little sequence conservation, their positioning within the middle region was conserved. The activation domain of ARF5/MP orthologs was centrally located, while those in the other class A ARF orthologs were located closer to either the DNA‐binding domain or PB1 domain. This conserved positioning, despite poor sequence conservation, suggests activation domain location influences functionality. However, its role in ARF‐co‐factor interactions and signaling specificity requires further investigation.

The PB1 domain not only connects ARFs to auxin regulation, it also plays a role in nearly all aspects of their function. The PB1 domain is a core element in auxin transcriptional regulation, but this functional diversity leaves several questions unanswered. Many have hypothesized specificity between ARF and Aux/IAA PB1 interactions; however, evidence has remained either circumstantial or predominantly limited to *in vitro* and heterologous assays (Berleth and Jürgens 1993; Hamann et al. 1999; Piya et al. 2014). Additional work is necessary to determine whether distinct ARF and Aux/IAA pairs regulate different sets of genes. Furthermore, the role of the PB1 domain in class B and C ARFs remains unclear, given their limited interactions with Aux/IAAs. ARF4 (class B), for example, interacts with nearly all Aux/IAAs (Piya et al. 2014) despite being relatively evolutionarily divergent (Hamm et al. 2019). Do Aux/IAA proteins interact with class B and C ARFs *in vivo* and, if so, do these interactions serve a functional purpose?

**FIGURE 7:**
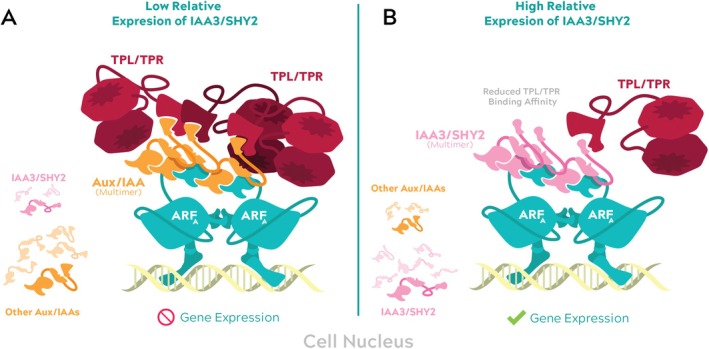
Aux/IAA‐mediated transcriptional specificity mediated by Aux/IAA affinity for TPL/TPR co‐repressors. Under low relative expression of IAA3/SHY2 (A), other Aux/IAAs interact with class A ARFs, recruiting TPL/TPR to block gene expression. Under high relative expression of IAA3/SHY2, it competes with other Aux/IAAs to interact with class A ARFs while having a much lower affinity for TPL/TPR, allowing gene expression to remain active.

Others have raised questions regarding the mechanism that underlies Aux/IAA‐facilitated repression. Currently, most of what is known about Aux/IAA interactions comes from *in vitro* experiments. Do these interactions hold up *in planta*? For example, Tao & Estelle (2018) found that, in *P. patens*, the EAR motif is not essential for Aux/IAA function. This suggests that Aux/IAAs possess some innate repressive activity. Furthermore, several Aux/IAAs appear to contain a second EAR motif in between Domains I and II or an extended motif (LxLxLxL or LxLxLxLxL) (Figure 7), although their impact on TPL/TPR recruitment and transcriptional repression remains unclear. In addition, multimerization of Aux/IAAs via the PB1 domain may be required in certain instances for ARF repression (Korasick et al. 2015), but Guilfoyle (2015) proposed that Aux/IAAs may alternatively need to interact with both the positive and negative face of a single ARF PB1 domain to achieve full repression. Further investigation is required to elucidate the exact structural mechanism in which multiple Aux/IAAs interact with ARF dimers.

**FIGURE 8:**
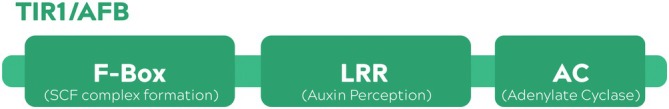
Schematic overview of three conserved regions found in TIR1/AFBs. N‐terminal F‐Box domain: Promotes interactions with other members of the SCF complex; LRR (leucine‐rich region): contains the auxin binding pocket enabling auxin‐dependent interactions with Aux/IAA proteins. C‐terminal adenylate cyclase (AC) motif: catalyzes the production of cAMP.

Finally, what are the biochemical mechanisms that underlie the functional differences observed in TIR1/AFBs? How does AFB1, which has been shown to mediate fast auxin response, do so while not appearing to interact with CUL1? How does homo‐oligomerization of TIR1 contribute to auxin‐induced degradation of Aux/IAA proteins? Do TIR1/AFBs play a role in regulating class B and C ARFs? What are the downstream effectors regulated by the recently identified AC activity of TIR1/AFBs? Addressing these questions will clarify how TIR1/AFBs orchestrate diverse and context‐specific auxin responses.

## 8 CONCLUDING REMARKS

At face value, the auxin signaling pathway constitutes a fairly straightforward mechanism for auxin perception and signaling. Nearly 100 years of auxin research have only begun to illuminate how the components of its signaling pathway can interact to facilitate diverse transcriptional responses to the generic auxin signal. Gradually, a framework to describe auxin signaling specificity is beginning to emerge.

Functional diversity in auxin signaling machinery family members allows for many combinatorial interactions to likely drive many distinct transcriptional responses. This highlights how ARFs' cell‐ and tissue‐specific expression and their distinct DNA‐binding preferences enable context‐dependent transcriptional responses of auxin‐regulated genes. The distinct functional roles of each of these components, further tuned by co‐expression patterns, post‐translational modifications, regulatory RNAs, and crosstalk with other pathways, bestow auxin signaling with dynamic tunability—controlling its sensitivity and specificity—and allowing for precise control over spatial and temporal responses.

Ultimately, answering outstanding questions and integration of all factors that contribute to auxin signaling specificity are necessary to fully understand how auxin mediates specific developmental responses. Advancements in artificial intelligence, high‐resolution imaging, synthetic biology, and bioinformatics, in combination with multi‐omic approaches, will undoubtedly help clarify the diverse auxin signals amongst all the noise.

## AUTHOR CONTRIBUTIONS

Joseph S. Taylor: conceptualization, writing‐original draft, figure design. Bastiaan O. R. Bargmann: conceptualization, writing‐review editing, supervision, funding acquisition.

## Data Availability

Not applicable.
